# On Simple Ulcer of the Stomach

**Published:** 1856-07

**Authors:** 


*On Simple Ulcer of the Stomach.*—By. M. Cruveilhier.

M. Cruveilhier has recently presented two papers to the Acadèmie des Sciences upon this subject, and the following are the general conclusions:—1. There exists a disease of the stomach that may be anatomically characterised as simple ulcer of the stomach, usually chronic. 2. This lesion, which is far more common than is usually supposed, differs from cancerous ulcer, with which it is generally confounded, in its curability. 3. It is susceptible of complete cicatrization, this being accomplished by means of very firm fibrous tissue, differing essentially from scirrhus, with which it has been confounded. 4. When the ulcer penetrates through the whole of the coats of the stomach, the loss of substance is repaired by surrounding organs, which also sometimes participate in the ulceration. 5. Danger may continue even after the cure of the ulcer, as the cicatrix often becomes the seat of consecutive ulceration, with all its attendant accidents. 6. It is one of the most frequent causes of blackish vomiting and dejections, and the most frequent one of hæmorrhage of the stomach, whether accompanied by hæmatemesis or not. 7. Simple ulcer is the most frequent cause of perforation of the stomach. 8. The two principal accidents are hæmorrhage and perforation, which take place more commonly consecutively- *i. e.*, by the erosion of the cicatrix, than primarily, or during the period of formation of the ulcer. 9. This ulcer, or ulcerative gastritis, may be always suspected, and almost always positively diagnosed. 10. It is distinguished from idiopathic gastralgia by the permanence of the symptoms it gives rise to, although these have alternations of exasperation and remission. Gastralgia is only temporary, comes and goes suddenly, leaving no traces of its presence, and may be suddenly relieved by opiates. 11. It is distinguished from non-ulcerative gastritis and gastralgia by black vomit and stools. It is very probable, however, that simple ulcer may exist without these discharges, and then its diagnosis from gastritis would be difficult. These black discharges are not characteristic of cancer; and, to some extent, are more inherent to simple ulcer than to it, for they belong to all periods of simple ulcer, of which they constitute the first symptom, while cancerous ulcer is not attended with them until the last stage, and sometimes not at all. 12. The distinctions between simple and cancerous ulcer are founded on, first, the physical signs, there being no tumour in the former; and, next, on the pain which is often absent in cancer but never in ulcer. The pain in the latter is like that of an open wound or burn, opposite the xyphoid appendix, striking through to the spine. In cancer there are cramps or spasmodic contractions, with induration of the stomach. 13. The true touchstone is the effect of alimentary regimen, which completely fails in cancer, but succeeds surprisingly in ulcer. 14. The great object in treating the disease is to find an aliment that is tolerated by the stomach without pain, for then the cure may soon be effected. In the immense majority of cases, milk diet induces improvement from the very first day, and sometimes operates like magic; but when it ceases to be agreeable to the patient, or fatigues the stomach, we must unite it with other substances, in the choice of which the instincts of the stomach must be consulted. Alimentary regimen, in fact, constitutes the entire treatment, but nothing can be more difficult than the direction of this, according to quantity, quality, repetition, preparation, and temperature. 15. Medicinal substances, whether general or topical, are quite secondary in importance. Iron and bitters are quite contra-indicated; and opium only succeeds when gastralgia is associated with the inflammatory action. Gaseous waters, ice, alkalis, and especially phosphate of lime prepared by the calcination of bone, alkaline and gelatinous baths, cold ablution of the entire surface, (in some cases very hot ablutions), cold baths, and, in some cases, very hot sitting baths, stimulant frictions, with shampooing of the entire surface, derivatives or revulsives applied to the epigastrium—are the means which have seemed to exert most influence on the progress of the disease. 16. It must never be forgotten, that this ulcer is very liable to relapse, such relapse sometimes going on to hæmorrhage or perforation. Such relapse may be certainly prevented by a good alimentary hygiene, and avoiding medicinal stimuli.—*Med. Times & Gaz. from Comptes Rendus.*

